# Iron and Virulence in *Francisella tularensis*

**DOI:** 10.3389/fcimb.2017.00107

**Published:** 2017-04-04

**Authors:** Girija Ramakrishnan

**Affiliations:** Department of Medicine/Division of Infectious Diseases, University of VirginiaCharlottesville, VA, USA

**Keywords:** Siderophore, FeoB, intracellular pathogen, FslE, FupA, TonB-independent, *Francisella tularensis*

## Abstract

*Francisella tularensis*, the causative agent of tularemia, is a Gram-negative bacterium that infects a variety of cell types including macrophages, and propagates with great efficiency in the cytoplasm. Iron, essential for key enzymatic and redox reactions, is among the nutrients required to support this pathogenic lifestyle and the bacterium relies on specialized mechanisms to acquire iron within the host environment. Two distinct pathways for iron acquisition are encoded by the *F. tularensis* genome- a siderophore-dependent ferric iron uptake system and a ferrous iron transport system. Genes of the Fur-regulated *fslABCDEF* operon direct the production and transport of the siderophore rhizoferrin. Siderophore biosynthesis involves enzymes FslA and FslC, while export across the inner membrane is mediated by FslB. Uptake of the rhizoferrin- ferric iron complex is effected by the siderophore receptor FslE in the outer membrane in a TonB-independent process, and FslD is responsible for uptake across the inner membrane. Ferrous iron uptake relies largely on high affinity transport by FupA in the outer membrane, while the Fur-regulated FeoB protein mediates transport across the inner membrane. FslE and FupA are paralogous proteins, sharing sequence similarity and possibly sharing structural features as well. This review summarizes current knowledge of iron acquisition in this organism and the critical role of these uptake systems in bacterial pathogenicity.

## Introduction

*Francisella tularensis*, the etiological agent of the zoonosis tularemia, is a Gram-negative gamma-proteobacterium with a small genome of 1.89 Mb (Sjöstedt, 2007). The species is further differentiated into three subspecies, of which *tularensis* causes a more severe disease than *holarctica*, while *mediasiatica* is less well-studied. The closely related species *F. novicida*, considered to be of a more ancestral lineage (Svensson et al., 2005) and with a genome sequence identity of ~98% (Larsson et al., 2009) is an opportunistic human pathogen, but can cause a virulent tularemia-like disease in mice.

In the laboratory, studies have largely focused on virulent strain Schu S4 of the *tularensis* subspecies, the attenuated live vaccine strain (LVS) derived from a *holarctica* isolate and the strain U112 of *F. novicida*. These three isolates share many biological attributes although their genetic and functional differences significantly impact virulence (Jones et al., 2012; Celli and Zahrt, 2013; Kingry et al., 2014). For this reason, all three strains are referred to in this review as *F. tularensis* unless specifically identified in order to highlight particular differences.

*F. tularensis* is a facultative intracellular pathogen infecting a wide variety of cells, including mammalian and arthropod cells (Ozanic et al., 2015). Following uptake into the macrophage, the bacteria at first reside within a phagosome, but then rapidly escape into the cytoplasm. Phagosomal escape is dependent on the *igl* operon and associated genes in the *Francisella* Pathogenicity Island (FPI) that encode components of a putative Type VI secretion system (Barker et al., 2009; de Bruin et al., 2011). The bacteria replicate to high numbers in the cytoplasm resulting finally in apoptotic death of the host cell. Adaptation to the specialized intracellular lifestyle is associated with evolutionary loss of genes for many metabolic pathways (Rohmer et al., 2007; Larsson et al., 2009), but *F. tularensis* has retained or evolved mechanisms to efficiently acquire essential nutrients within the intracellular niche of the different cell types that it infects (Meibom and Charbit, 2010).

Mice have been extensively used to model animal infection (Lyons and Wu, 2007). Phagocytic cells are thought to be the first infected (Hall et al., 2008); subsequently infection is disseminated to other tissues in the body. *F. tularensis* exercises several strategies to evade immune responses and is able to replicate to high levels in the liver, spleen and lungs before the immune system is provoked to respond with a destructive cytokine storm (Sharma et al., 2011; Jones et al., 2012).

### Iron and francisella

*Francisella* requires iron for essential cellular functions. Early studies reported that infection with *F. tularensis* induces an iron-withholding response typical of the innate nutritional immunity defense mechanism (Pekarek et al., 1969). However, the intracellular pathogen manipulates host cell iron metabolism to support growth; LVS induces infected macrophages to increase iron flow through the cell by enhanced expression of the transferrin receptor TfR1 for uptake of iron and in parallel, increased expression of Dmt1 that moves endosomal iron into the cytoplasm and a slight increase in ferroportin that promotes outflow of iron from the cell (Pan et al., 2010). A functional Nramp1protein that also transports endosomal iron into the cytoplasm restricts growth of endosome-resident bacteria but enhances *Francisella* growth (Kovářová et al., 2000, 2002), highlighting the importance of cytoplasmic iron availability for pathogenesis. However, the nature of the host iron sources accessed by the organism remains to be characterized. Iron-limitation restricts growth of bacteria in culture (Deng et al., 2006; Sullivan et al., 2006) as well as within the macrophage (Fortier et al., 1995).

As might be predicted, growth of *F. tularensis* is inhibited by gallium, which competes with ferric iron for uptake and also interferes with iron-dependent biological processes (Olakanmi et al., 2010; Lindgren and Sjöstedt, 2016). Inhibition of the iron-associated enzymes catalase and superoxide dismutase leads to increased susceptibility to oxidative stress (Bakshi et al., 2006; Lindgren et al., 2007; Olakanmi et al., 2010; Binesse et al., 2015).

Iron metabolism appears to differ among *F. tularensis* isolates. LVS expresses higher levels of bacterioferritin as compared to Schu S4 (Hubálek et al., 2003, 2004). Consistent with these findings, isolates of the *holarctica* subspecies have greater iron stores than *tularensis* isolates, and since iron is closely associated with generation of reactive oxygen species, *holarctica* strains are more susceptible to oxidative stress (Lindgren et al., 2011).

### Fur and iron regulation of genes

**Figure 1:**
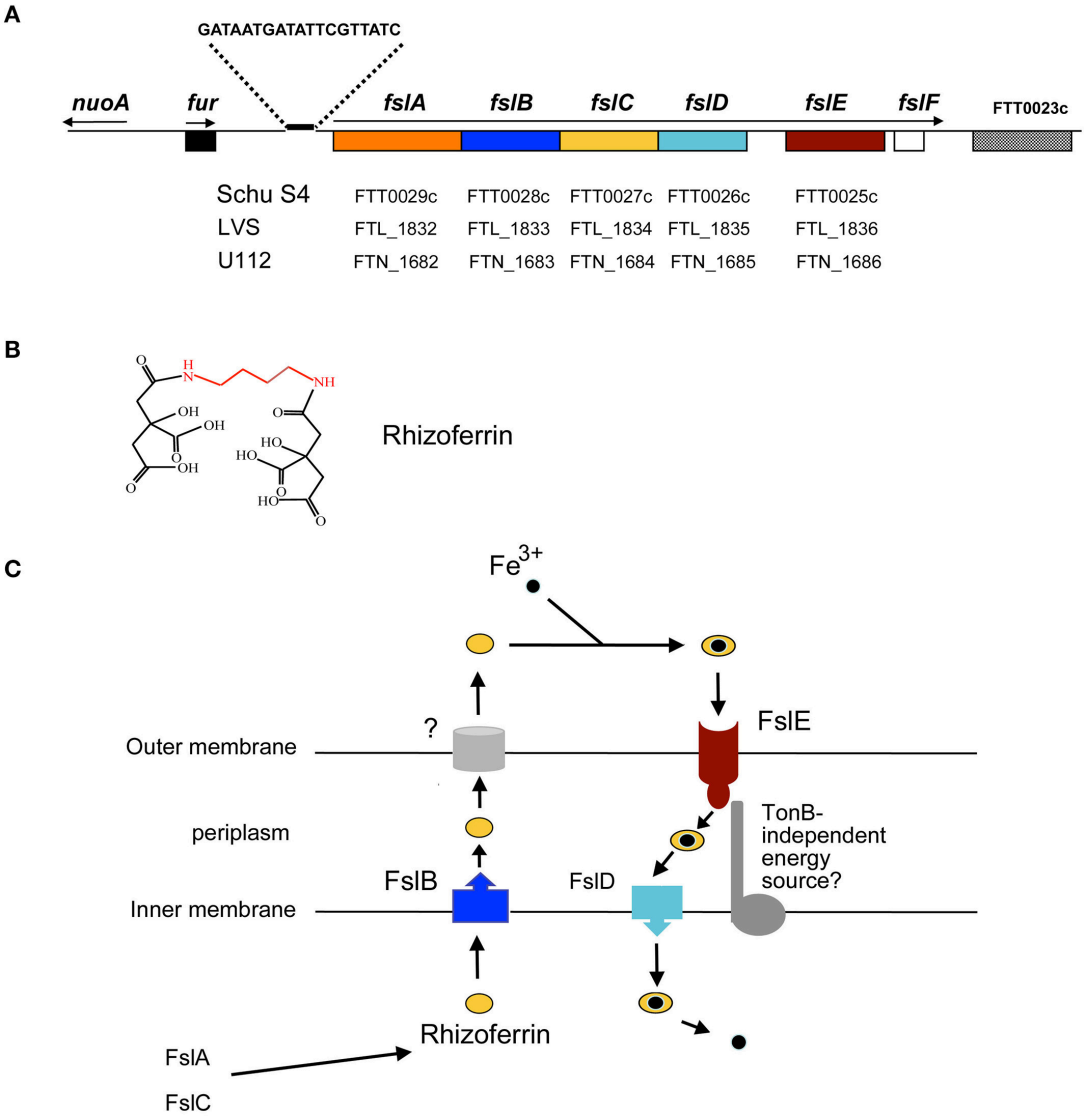
**Siderophore-mediated iron acquisition in ***F. tularensis***. (A)** The *fur-fsl* locus of the *F. tularensis* subsp. *tularensis* chromosome is depicted and the corresponding gene designations in the Schu S4, LVS, and U112 genomes are indicated. The Furbox upstream of the *fslABCDEF* operon is shown. Arrows indicate the direction and extent of transcribed regions. **(B)** A schematic of the *F. tularensis* siderophore, rhizoferrin comprising two citrate molecules linked by amide bonds to a putrescine backbone (in red). **(C)** Current model for siderophore-mediated iron acquisition in *F. tularensis* and the roles of the *fsl* operon products. FslA and FslC encode enzymes for biosynthesis of the siderophore. The siderophore is released into the extracellular medium by the action of FslB and an as yet unknown outer membrane component. The outer membrane siderophore receptor FslE, and FslD in the inner membrane mediate uptake of the ferric-siderophore complex. It is not known if a TonB analog facilitates FslE function.

A *fur* ortholog is encoded in the *F. tularensis* genome and the predicted Fur protein contains elements known to be important for Fur function (Pérard et al., 2016). The *fur* gene is adjacent to the *fsl* operon encoding components of a siderophore- mediated iron uptake pathway, and a canonical Furbox is located upstream of the first gene of the operon, *fslA* (Figure 1A). Expression of the *fsl* operon is induced in iron-limiting media (Deng et al., 2006; Sullivan et al., 2006; Buchan et al., 2008). Loss of the *fur* gene results in deregulated transcription of the *fsl* operon and increased siderophore production (Buchan et al., 2008; Ramakrishnan et al., 2008). Expression of the inner membrane ferrous iron transporter *feoB* is also upregulated in a *fur* mutant (Pérez and Ramakrishnan, 2014).

Exposure to iron limitation was also shown to increase virulence of an *F. tularensis* isolate, suggesting that pathogenicity is influenced by iron levels (Bhatnagar et al., 1995). Microarray analysis of RNA indicated that besides the *fsl* operon, transcription of the *igl* operon was increased under iron limitation (Deng et al., 2006). Proteomic analysis also confirmed that iron limitation results in increased levels of the IglC protein (Lenco et al., 2007). However, although an *fslB-lacZ* reporter could be repressed by overexpression of Fur, an *iglB-lacZ* reporter was not similarly repressible (Buchan et al., 2008), suggesting that a mechanism besides Fur regulates the *igl* genes in response to iron levels in *F. tularensis*.

### Siderophore-mediated iron acquisition

A “growth inducing substance (GIS)” that promoted growth of *F. tularensis* bacteria from small inocula was reported in the 1960s (Halmann and Mager, 1967; Halmann et al., 1967); in all likelihood this substance was the siderophore now identified as the polycarboxylate rhizoferrin (Drechsel et al., 1991; Thieken and Winkelmann, 1992; Sullivan et al., 2006). Rhizoferrin is structurally simple, comprising 2 citrate moieties linked through amide bonds to a putrescine backbone (Figure 1B). Originally identified as a fungal siderophore (Drechsel et al., 1991; Thieken and Winkelmann, 1992), rhizoferrin was subsequently also isolated from a strain of the bacterium *Ralstonia pickettii* (Münzinger et al., 1999). The identification of the siderophore made by *F. tularensis* as rhizoferrin (Sullivan et al., 2006), and the subsequent identification of the *Legionella pneumophila* siderophore legiobactin as also rhizoferrin (Burnside et al., 2015) suggests that this siderophore may be more widely prevalent in bacteria than suspected. Structurally related siderophores made by bacteria include staphyloferrin A (Konetschny-Rapp et al., 1990) and corynebactin (Zajdowicz et al., 2012), where D-ornithine and lysine, respectively, constitute the siderophore backbone in place of the putrescine present in rhizoferrin. The *R. pickettii* rhizoferrin was shown by CD spectroscopy to be an S-S enantiomer in contrast to the R-R fungal molecule (Münzinger et al., 1999). Whether all bacterial rhizoferrins adopt the S-S conformation is not clear, but bacteria making rhizoferrin are capable of utilizing the fungal form for iron uptake (Münzinger et al., 1999; Kiss et al., 2008).

Genes for synthesis and transport of *Francisella* rhizoferrin are located on the siderophore operon *fslABCDEF* (also designated *figABCDEF*; (Deng et al., 2006; Sullivan et al., 2006; Milne et al., 2007; Buchan et al., 2008; Ramakrishnan et al., 2008); Figures 1A,C). Analysis of individual mutants as well as complementation of a strain carrying a complete deletion of the *fslA-F* genes helped to determine the roles played by the different genes in siderophore-mediated iron acquisition, as detailed below.

*fslA* and *fslC* and share homology with genes found in siderophore biosynthetic loci of other bacteria. FslA is similar to the aerobactin synthetases IucA/IucC and a member of the non-ribosomal peptide synthetase-independent siderophore (NIS) synthetases, enzymes that assemble non-peptide siderophores using dicarboxylic acids and diamines or amino-alcohols (Challis, 2005). FslC is predicted to be a member of the pyridoxal phosphate-dependent decarboxylases. Mutant analysis demonstrated that both *fslA* and *fslC* are required for *Francisella* rhizoferrin production (Deng et al., 2006; Sullivan et al., 2006; Lindgren et al., 2009; Thomas-Charles et al., 2013). Rhizoferrin biosynthesis in *F. tularensis*, involving just two dedicated enzymes, may be the simplest siderophore biosynthetic pathway identified thus far.

*fslB* encodes a transporter of the Major Facilitator superfamily (MFS) and deletion of this gene in *F. novicida* results in reduced levels of siderophore activity in the culture medium (Kiss et al., 2008). Additionally, detection of siderophore activity in culture supernatants of an LVS Δ*fslA-F* mutant required complementation with the *fslB* gene in addition to the biosynthetic genes *fslA* and *fslC* (Pérez et al., 2016). These observations support a role for FslB in export of the siderophore across the cytoplasmic membrane. How the siderophore is channeled through the outer membrane into the extracellular space is currently not known.

*fslD* encodes an inner membrane MFS protein and deletion of this gene was found to have little effect on siderophore production (Kiss et al., 2008). The role of this protein in siderophore-mediated iron uptake across the cytoplasmic membrane was deduced on the basis of complementation studies: a Schu S4 Δ*fslA-F* mutant is able to transport ^55^Fe^3+^ complexed to siderophore only when complemented with the *fslD* gene in addition to the *fslE* receptor gene (see below; Pérez et al., 2016).

*fslE*, the fifth gene in the operon, encodes an outer membrane protein unique to the *Francisella* genus Larsson et al., 2005; Huntley et al., 2007). Δ*fslE* mutants are impaired for growth in iron-limiting media and are unable to utilize exogenous siderophore for growth (Kiss et al., 2008; Ramakrishnan et al., 2008). In transport assays, *fslE* mutants proved incapable of siderophore-mediated ^55^Fe^3+^ uptake, establishing a role for FslE as receptor for the siderophore (Ramakrishnan et al., 2012). FslE can also transport the iron mimic gallium in complex with rhizoferrin as shown by the resistance of *fslA* and *fslE* mutants to gallium (Pérez et al., 2016).

The last gene of the *fsl* operon, *fslF*, varies structurally among the *tularensis* and the *novicida* species, being truncated in the *tularensis* isolates. Studies with a Δ*fslF* mutant in Schu S4 indicate that the gene does not influence iron transport in *F. tularensis* (Pérez et al., 2016).

Siderophore-mediated iron transport by outer membrane receptors in Gram-negative bacteria is typically dependent on the proton motive force transduced by the TonB-ExbB-ExbD complex (Noinaj et al., 2010). The *Francisella* genome, however, does not encode orthologs of *tonB, exbB*, and *exbD*, implying that alternative mechanisms must facilitate siderophore -iron uptake.

### Ferrous iron uptake

**Figure 2:**
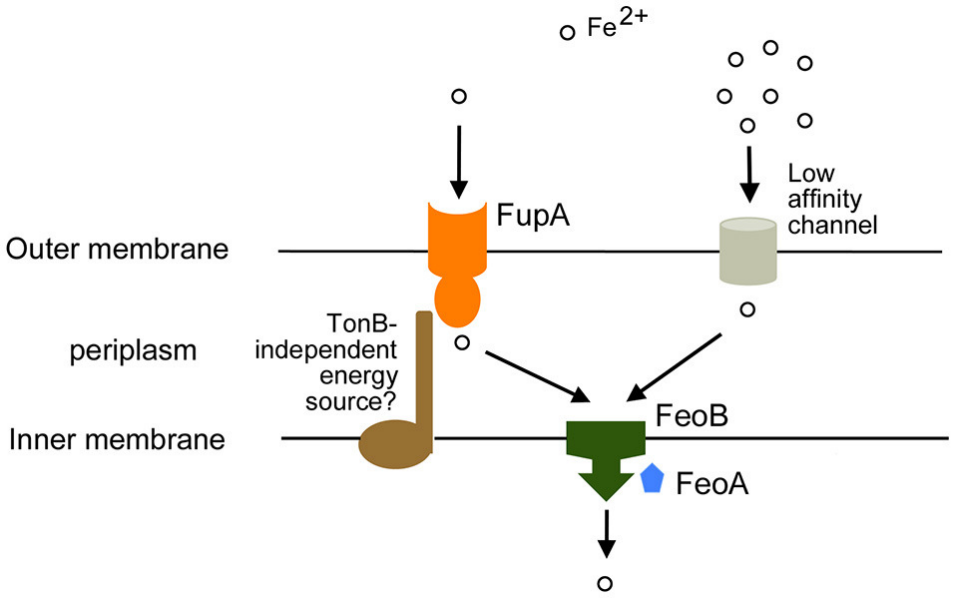
**Ferrous iron acquisition in ***F. tularensis*****. Transport of ferrous iron relies on the high affinity transporter FupA and uncharacterized low affinity channels in the outer membrane. Inner membrane transport is dependent on the FeoB transporter with likely involvement of FeoA. We raise the possibility that an accessory protein may facilitate FupA function.

The *F. tularensis* genome encodes an inner membrane ferrous iron transport system comprising unlinked genes *feoA* and *feoB*. Δ*feoB* mutants of LVS and Schu S4 are deficient for growth on iron-limiting media (Thomas-Charles et al., 2013; Pérez and Ramakrishnan, 2014; Pérez et al., 2016). ^55^Fe uptake assays demonstrated that the *F. tularensis* Δ*feoB* mutants are completely deficient in ferrous iron uptake (Pérez and Ramakrishnan, 2014; Pérez et al., 2016), implying that the Feo system is the sole ferrous iron transporter across the inner membrane (Figure 2). It is likely that FeoA supports FeoB function as seen in *Salmonella* Typhimurium (Kim et al., 2012) and *Vibrio cholerae* (Weaver et al., 2013; Stevenson et al., 2016).

Given the soluble nature of ferrous iron, the general assumption has been that it diffuses into the periplasmic space through non-specific porin proteins in the outer membrane. However, growth and ^55^Fe transport assays indicate that *F. tularensis* is capable of high-affinity uptake of ferrous iron mediated by the specific outer membrane protein FupA (Ramakrishnan et al., 2012). FupA was initially characterized as a virulence factor in Schu S4 (Twine et al, 2005) and found to influence bacterial intracellular replication (Twine et al, 2005; Asare et al., 2010). An involvement in iron acquisition was established by the finding that Schu S4 Δ*fupA* mutant grew poorly under iron limitation, had lowered internal iron levels and was deregulated for siderophore production (Lindgren et al., 2009). A link to ferrous iron uptake was indicated by the finding that ferrous and ferric iron supplements supported growth of a *fupA* mutant to different extents on agar plates (Ramakrishnan et al., 2012). ^55^Fe transport assays clearly demonstrated that the *fupA* mutant was unable to transport ferrous iron at limiting concentrations (~0.1 μM) although low affinity ferrous iron transport at high concentrations (~3 μM) could still be observed, and siderophore-iron uptake was not perturbed (Ramakrishnan et al., 2012). These findings suggest that FupA serves as a *F*. *tularensis* adaptation to efficiently acquire ferrous iron even in low abundance settings when general diffusion-based transport across the outer membrane might prove inadequate (Figure 2).

### FslE and FupA: related high-affinity iron transport proteins

FupA and FslE are paralogs belonging to a family of proteins unique to *Francisella* (Larsson et al., 2005). Both proteins have been localized to the outer membrane of *F. tularensis* (Huntley et al., 2007; Ramakrishnan and Sen, 2014) and share a global 54% identity and 69% similarity in amino acid sequence. FupA with 557 amino acid residues is larger than FslE (509 residues). The Hidden Markov Model-based PRED-TMBB program (Bagos et al., 2004a,b) predicts that both FupA and FslE fold as β-barrels in the outer membrane with amino-terminal periplasmic domains. FupA is predicted to form a 16-stranded barrel with a periplasmic domain of 201 residues while FslE could form a 14-stranded barrel with a 152 residue periplasmic domain. This structure is reminiscent of typical TonB-dependent transporters (Noinaj et al., 2010). The greatest similarity between FslE and FupA is in the predicted β-barrel domains.

The *fupA* gene is located adjacent to a paralog *fupB* on the chromosome. Infrequent recombinational deletion events have been observed leading to formation of *fupA/B* hybrid genes (Twine et al, 2005; Rohmer et al., 2006); such recombination accounts for a significant reduction in virulence as seen in LVS, which can be reversed by restoration of the full length *fupA* gene (Salomonsson et al., 2009). The FupA/B hybrid protein encoded by LVS is less efficient at high-affinity ferrous iron uptake than FupA, but gains siderophore-iron uptake capability (Sen et al., 2010; Ramakrishnan and Sen, 2014). The structural and functional overlap in protein function raises the intriguing possibility that a common mechanism may underlie transport by FslE and FupA.

*fupA* expression is independent of iron and *fur* regulation, suggesting that FupA may have functions in addition to iron transport. *fupA* mutants have increased resistance to copper, and transport assays indicated that copper competes with ferrous iron for transport (Pérez et al., 2016). A role for FupA in maintenance of outer membrane integrity has also been proposed (Nallaparaju et al., 2011). Alhough, the *fsl* operon is regulated in response to iron levels, it was reported recently that calcium and magnesium limitation also result in increased *fslE* transcription (Wu et al., 2016). These observations are consistent with the idea that the high affinity iron transport proteins in the outer membrane of *F. tularensis* may assume roles in transport of additional substrates under stress.

Interestingly, FslE appears structurally different from rhizoferrin receptors of other bacteria. The LbtU siderophore receptor of *L. pneumophila* is not predicted to have a distinct periplasmic domain (Chatfield et al., 2011). RumA, the rhizoferrin receptor in *Morganella morganii* is a TonB-dependent transporter (Kühn et al., 1996). Mechanisms for rhizoferrin transport thus appear to have evolved independently in different bacteria.

### Iron uptake and pathogenesis

Analysis of the transcriptome of Schu S4-infected macrophages demonstrated that the *fsl* genes are among the most highly induced genes in the intracellular niche (Wehrly et al., 2009). Similar results were obtained using LVS infected hepatocytes (Thomas-Charles et al., 2013), suggesting that the siderophore uptake system contributes to survival within different tissue types. *fsl* mutants were identified in a negative selection screen of a U112 transposon mutant library in mice (Weiss et al., 2007) and a signature-tagged mutant screen identified *fslA* and *feoB* to be important for pulmonary infection by LVS (Su et al., 2007). Nevertheless, individual mutants in the *fsl, fupA*, and *feoB* genes are capable of intracellular growth although the reduced growth of the *feoB* mutants in hepatocytes suggests that ferrous iron is likely the major iron source within these cells (Lindgren et al., 2009; Ramakrishnan et al., 2012; Thomas-Charles et al., 2013; Pérez and Ramakrishnan, 2014; Pérez et al., 2016). Both iron uptake pathways appear to contribute to utilization of iron from heme for growth (Lindgren et al., 2015), suggesting that iron needs to be released from the heme for use by the bacteria. Screens with U112 mutants have implicated additional genes in iron acquisition, but they have not been definitively characterized (Crosa et al., 2009).

A Schu Δ*fslE* Δ*fupA* mutant deficient for both siderophore and high affinity ferrous iron uptake grows slowly, is attenuated for growth in macrophages and completely avirulent in mice (Ramakrishnan et al., 2012). Δ*fslA* Δ*feoB* mutants of LVS and Schu S4 deficient for siderophore biosynthesis and for all ferrous iron uptake have an even more severe defect, with dependence on extraneous siderophore for growth, loss of all capacity for intracellular growth and complete loss of virulence (Pérez and Ramakrishnan, 2014; Pérez et al., 2016). These findings indicate that both subspecies of *F. tularensis* have a similar repertoire of iron uptake mechanisms, limited to just the *fsl* and *feo-*mediated mechanisms. Interestingly, although the Δ*fslE* Δ*fupA* and the Δ*fslA* Δ*feoB* mutants are avirulent in mice, exposure to these strains protects from subsequent challenge with the virulent strain (Ramakrishnan et al., 2012; Pérez et al., 2016), making them good candidates for further exploration as live vaccines.

## Conclusions

With a reduced genome, *F. tularensis* has evolved to efficiently support its lifestyle as an intracellular pathogen with a minimal set of two iron acquisition pathways. Host iron sources utilized and mechanisms regulating the transport proteins FslE and FupA are interesting questions for future investigations.

## Author contributions

The author confirms being the sole contributor of this work and approved it for publication.

## Funding

Work in the author's lab has been supported by grants AI056227, AI067823, and AI119471 from the National Institute of Allergy and Infectious Diseases (NIH) and by intramural support from the School of Medicine, University of Virginia.

### Conflict of interest statement

The author declares that the research was conducted in the absence of any commercial or financial relationships that could be construed as a potential conflict of interest.

## References

[B1] Asare R. Akimana C. Jones S. Abu Kwaik Y. (2010). Molecular bases of proliferation of *Francisella tularensis* in arthropod vectors. Environ. Microbiol. 12, 2587–2612. 10.1111/j.1462-2920.2010.02230.x 20482589 PMC2957557

[B2] Bagos P. G. Liakopoulos T. D. Spyropoulos I. C. Hamodrakas S. J. (2004a). A hidden markov model method, capable of predicting and discriminating β barrel outer membrane proteins. BMC Bioinformatics 5:29. 10.1186/1471-2105-5-29 15070403 PMC385222

[B3] Bagos P. G. Liakopoulos T. D. Spyropoulos I. C. Hamodrakas S. J. (2004b). PRED-TMBB: a web server for predicting the topology of β barrel outer membrane proteins. Nucleic Acids Res. 32, W400–W404. 10.1093/nar/gkh417 15215419 PMC441555

[B4] Bakshi C. S. Malik M. Regan K. Melendez J. A. Metzger D. W. Pavlov V. M.. (2006). Superoxide dismutase B gene (sodB)-deficient mutants of *Francisella tularensis* demonstrate hypersensitivity to oxidative stress and attenuated virulence. J. Bacteriol. 188, 6443–6448. 10.1128/JB.00266-06 16923916 PMC1595384

[B5] Barker J. R. Chong A. Wehrly T. D. Yu J. J. Rodriguez S. A. Liu J.. (2009). The *Francisella tularensis* pathogenicity island encodes a secretion system that is required for phagosome escape and virulence. Mol. Microbiol. 74, 1459–1470. 10.1111/j.1365-2958.2009.06947.x 20054881 PMC2814410

[B6] Bhatnagar N. B. Elkins K. L. Fortier A. H. (1995). Heat stress alters the virulence of a rifampin-resistant mutant of *Francisella tularensis* LVS. Infect. Immun. 63, 154–159. 7806352 10.1128/iai.63.1.154-159.1995 PMC172972

[B7] Binesse J. Lindgren H. Lindgren L. Conlan W. Sjöstedt A. (2015). Roles of reactive oxygen species-degrading enzymes of *Francisella tularensis* SCHU S4. Infect. Immun. 83, 2255–2263. 10.1128/IAI.02488-14 25802058 PMC4432764

[B8] Buchan B. W. McLendon M. K. Jones B. D. (2008). Identification of differentially regulated *Francisella tularensis* genes by use of a newly developed Tn*5*-based transposon delivery system. Appl. Environ. Microbiol. 74, 2637–2645. 10.1128/AEM.02882-07 18344342 PMC2394869

[B9] Burnside D. M. Wu Y. Shafaie S. Cianciotto N. P. (2015). The *Legionella pneumophila* siderophore legiobactin is a polycarboxylate that is identical in structure to rhizoferrin. Infect. Immun. 83, 3937–3945. 10.1128/IAI.00808-15 26195554 PMC4567642

[B10] Celli J. Zahrt T. C. (2013). Mechanisms of *Francisella tularensis* intracellular pathogenesis. Cold Spring Harb. Perspect. Med. 3:a010314. 10.1101/cshperspect.a010314 23545572 PMC3683997

[B11] Challis G. L. (2005). A widely distributed bacterial pathway for siderophore biosynthesis independent of nonribosomal peptide synthetases. ChemBioChem 6, 601–611. 10.1002/cbic.200400283 15719346

[B12] Chatfield C. H. Mulhern B. J. Burnside D. M. Cianciotto N. P. (2011). *Legionella pneumophila* LbtU acts as a novel, tonb-independent receptor for the legiobactin siderophore. J. Bacteriol. 193, 1563–1575. 10.1128/JB.01111-10 21278293 PMC3067665

[B13] Crosa L. M. Crosa J. H. Heffron F. (2009). Iron Transport in *Francisella* in the absence of a recognizable TonB protein still requires energy generated by the proton motive force. Biometals 22, 337–344. 10.1007/s10534-008-9170-7 18946633

[B14] de Bruin O. M. Duplantis B. N. Ludu J. S. Hare R. F. Nix E. B. Schmerk C. L.. (2011). The biochemical properties of the Francisella Pathogenicity Island (FPI)-encoded proteins IglA, IglB, IglC, PdpB and DotU Suggest roles in Type VI secretion. Microbiology 157, 3483–3491. 10.1099/mic.0.052308-0 21980115 PMC3352279

[B15] Deng K. Blick R. J. Liu W. Hansen E. J. (2006). Identification of *Francisella tularensis* genes affected by iron limitation. Infect. Immun. 74, 4224–4236. 10.1128/IAI.01975-05 16790797 PMC1489736

[B16] Drechsel H. Metzger J. Freund S. Jung G. Boelaert J. R. Winkelmann G. (1991). Rhizoferrin- a novel siderophore from the fungus *Rhizopus microsporus* Var. *Rhizopodiformis*. Biometals 4, 238–243.

[B17] Fortier A. H. Leiby D. A. Narayanan R. B. Asafoadjei E. Crawford R. M. Nacy C. A.. (1995). Growth of *Francisella tularensis* LVS in Macrophages: the acidic intracellular compartment provides essential iron required for growth. Infect. Immun. 63, 1478–1483. 7890413 10.1128/iai.63.4.1478-1483.1995 PMC173178

[B18] Hall J. D. Woolard M. D. Gunn B. M. Craven R. R. Taft-Benz S. Frelinger J. A. (2008). Infected-host-cell repertoire and cellular response in the lung following inhalation of *Francisella tularensis* Schu S4, LVS, or U112. Infect. Immun. 76, 5843–5852. 10.1128/IAI.01176-08 18852251 PMC2583552

[B19] Halmann M. Mager J. (1967). An endogenously produced substance essential for growth initiation of *Pasteurella tularensis*. J. Gen. Microbiol. 49, 461–468. 10.1099/00221287-49-3-461

[B20] Halmann M. Magda B. Mager J. (1967). Nutritional requirements of *Pasteurella tularensis* for growth from small inocula. J. Gen. Microbiol. 49, 451–460. 10.1099/00221287-49-3-451

[B21] Hubálek M. Hernychová L. Brychta M. Lenčo J. Zechovská J. Stulík J. (2004). Comparative proteome analysis of cellular proteins extracted from highly virulent *Francisella tularensis* ssp. tularensis and less virulent, *F. tularensis* ssp. holarctica and *F. tularensis* ssp. mediaasiatica. Proteomics 4, 3048–3060. 10.1002/pmic.200400939 15378745

[B22] Hubálek M. Hernychová L. Havlasová J. Kasalová I. Neubauerová V. Stulík J.. (2003). Towards proteome database of *Francisella tularensis*. J. Chromatogr. B Analyt. Technol. Biomed. Life Sci. 787, 149–177. 10.1016/S1570-0232(02)00730-4 12659739

[B23] Huntley J. F. Conley P. G. Hagman K. E. Norgard M. V. (2007). Characterization of *Francisella tularensis* outer membrane proteins. J. Bacteriol. 189, 561–574. 10.1128/JB.01505-06 17114266 PMC1797401

[B24] Jones C. L. Napier B. A. Sampson T. R. Llewellyn A. C. Schroeder M. R. Weiss D. S. (2012). Subversion of host recognition and defense systems by *Francisella* spp. Microbiol. Mol. Biol. Rev. 76, 383–404. 10.1128/MMBR.05027-11 22688817 PMC3372254

[B25] Kim H. Lee H. Shin D. (2012). The FeoA protein is necessary for the FeoB transporter to import ferrous iron. Biochem. Biophys. Res. Commun. 423, 733–738. 10.1016/j.bbrc.2012.06.027 22705302

[B26] Kingry L. C. Petersen J. M. (2014). Comparative review of *Francisella tularensis* and *Francisella novicida*. Front. Cell. Infect. Microbiol. 4:35. 10.3389/fcimb.2014.00035 24660164 PMC3952080

[B27] Kiss K. Liu W. Huntley J. F. Norgard M. V. Hansen E. J. (2008). Characterization of fig operon mutants of *Francisella novicida* U112. FEMS Microbiol. Lett. 285, 270–277. 10.1111/j.1574-6968.2008.01237.x 18564336 PMC2770140

[B28] Konetschny-Rapp S. Jung G. Meiwes J. Zähner H. (1990). Staphyloferrin a: a structurally new siderophore from staphylococci. Eur. J. Biochem. 191, 65–74. 10.1111/j.1432-1033.1990.tb19094.x 2379505

[B29] Kovářová H. Halada P. Man P. Golovliov I. Kovářová Z. Špaček J.. (2002). Proteome Study of *Francisella tularensis* Live vaccine strain-containing phagosome in *Bcg/Nramp1* congenic macrophages: resistant allele contributes to permissive environment and susceptibility to infection. Proteomics 2, 85–93. 10.1002/1615-9861(200201)2:1<85::AID-PROT85>3.0.CO;2-S 11788995

[B30] Kovářová H. Hernychova L. Hajduch M. Sirova M. Macela A. (2000). Influence of the *Bcg* locus on natural resistance to primary infection with the facultative intracellular bacterium *Francisella tularensis* in mice. Infect. Immun. 68, 1480–1484. 10.1128/IAI.68.3.1480-1484.2000 10678963 PMC97304

[B31] Kühn S. Braun V. Köster W. (1996). Ferric rhizoferrin uptake into *Morganella morganii*: characterization of genes involved in the uptake of a polyhydroxycarboxylate siderophore. J. Bacteriol. 178, 496–504. 10.1128/jb.178.2.496-504.1996 8550472 PMC177684

[B32] Larsson P. Elfsmark D. Svensson K. Wikström P. Forsman M. Brettin T.. (2009). Molecular evolutionary consequences of niche restriction in *Francisella tularensis*, a facultative intracellular pathogen. PLoS Pathog. 5:e1000472. 10.1371/journal.ppat.1000472 19521508 PMC2688086

[B33] Larsson P. Oyston P. C. Chain P. Chu M. C. Duffield M. Fuxelius H. H.. (2005). The complete genome sequence of *Francisella tularensis*, the causative agent of tularemia. Nat. Genet. 37, 153–159. 10.1038/ng1499 15640799

[B34] Lenco J. Hubalek M. Larsson P. Fucikova A. Brychta M. Macela A.. (2007). Proteomics analysis of the *Francisella tularensis* LVS response to iron restriction: induction of the *F*. tularensis pathogenicity island proteins IglABC. FEMS Microbiol. Lett. 269, 11–21. 10.1111/j.1574-6968.2006.00595.x 17227466

[B35] Lindgren H. Sjöstedt A. (2016). Gallium potentiates the antibacterial effect of gentamicin against *Francisella tularensis*. Antimicrob. Agents Chemother. 60, 288–295. 10.1128/AAC.01240-15 26503658 PMC4704213

[B36] Lindgren H. Honn M. Golovlev I. Kadzhaev K. Conlan W. Sjostedt A. (2009). The 58-kilodalton major virulence factor of *Francisella tularensis* Is required for efficient utilization of iron. Infect. Immun. 77, 4429–4436. 10.1128/IAI.00702-09 19651867 PMC2747937

[B37] Lindgren H. Honn M. Salomonsson E. Kuoppa K. Forsberg Å. Sjö¨stedt A. (2011). Iron content differs between *Francisella tularensis Subspecies tularensis* and *Subspecies holarctica* strains and correlates to their susceptibility to H(2)O(2)-induced killing. Infect. Immun. 79, 1218–1224. 10.1128/IAI.01116-10 21189323 PMC3067496

[B38] Lindgren H. Lindgren L. Golovliov I. Sjöstedt A. (2015). Mechanisms of heme utilization by *Francisella tularensis*. PLoS ONE 10:e0119143. 10.1371/journal.pone.0119143 25756756 PMC4355490

[B39] Lindgren H. Shen H. Zingmark C. Golovliov I. Conlan W. Sjöstedt A. (2007). Resistance of *Francisella tularensis* strains against reactive nitrogen and oxygen species with special reference to the role of KatG. Infect. Immun. 75, 1303–1309. 10.1128/IAI.01717-06 17210667 PMC1828546

[B40] Lyons R. C. Wu T. H. (2007). Animal models of *Francisella tularensis* infection. Ann. N. Y. Acad. Sci. 1105, 238–265. 10.1196/annals.1409.003 17395735

[B41] Meibom K. L. Charbit A. (2010). *Francisella tularensis* metabolism and its relation to virulence. Front. Microbiol. 1:140. 10.3389/fmicb.2010.00140 21687763 PMC3109416

[B42] Milne T. S. Michell S. L. Diaper H. Wikström P. Svensson K. Oyston P. C.. (2007). A 55 kDa hypothetical membrane protein is an iron-regulated virulence factor of *Francisella tularensis* subsp. Novicida U112. J. Med. Microbiol. 56(Pt 10), 1268–1276. 10.1099/jmm.0.47190-0 17893160

[B43] Münzinger M. Taraz K. Budzikiewicz H. Drechsel H. Heymann P. Winkelmann G. (1999). S,S-Rhizoferrin (Enantio-Rhizoferrin) - a siderophore of *Ralstonia* (*Pseudomonas*) *pickettii* DSM 6297 - the optical antipode of R,R-rhizoferrin isolated from fungi. Biometals 12, 189–193. 10.1023/A:1009259118034

[B44] Nallaparaju K. C. Yu J. J. Rodriguez S. A. Zogaj X. Manam S. Guentzel M. N. Seshu J.. (2011). Evasion of IFN- γ signaling by *Francisella novicida* is dependent upon *Francisella* outer membrane protein, C. PLoS ONE 6:e18201. 10.1371/journal.pone.0018201 21483828 PMC3069069

[B45] Noinaj N. Guillier M. Barnard T. J. Buchanan S. K. (2010). TonB-dependent transporters: regulation, structure, and function. Annu. Rev. Microbiol. 64, 43–60. 10.1146/annurev.micro.112408.134247 20420522 PMC3108441

[B46] Olakanmi O. Gunn J. S. Su S. Soni S. Hassett D. J. Britigan B. E. (2010). Gallium disrupts iron uptake by intracellular and extracellular *Francisella* strains and exhibits therapeutic efficacy in a murine pulmonary infection model. Antimicrob. Agents Chemother. 54, 244–253. 10.1128/AAC.00655-09 19917753 PMC2798485

[B47] Ozanic M. Marecic V. Abu Kwaik Y. Santic M. (2015). The divergent intracellular lifestyle of *Francisella tularensis* in evolutionarily distinct host cells. PLoS Pathog. 11:e1005208. 10.1371/journal.ppat.1005208 26633893 PMC4669081

[B48] Pan X. Tamilselvam B. Hansen E. J. Daefler S. (2010). Modulation of iron homeostasis in macrophages by bacterial intracellular pathogens. BMC Microbiol. 10:64. 10.1186/1471-2180-10-64 20184753 PMC2838877

[B49] Pekarek R. S. Bostian K. A. Bartelloni P. J. Calia F. M. Beisel W. R. (1969). The effects of *Francisella tularensis* infection on iron metabolism in man. Am. J. Med. Sci. 258, 14–25. 10.1097/00000441-196907000-00003 4894492

[B50] Pérard J. Covés J. Castellan M. Solard C. Savard M. Miras R. Galop S.. (2016). Quaternary structure of fur proteins, a new subfamily of tetrameric proteins. Biochemistry 55, 1503–1515. 10.1021/acs.biochem.5b01061 26886069

[B51] Pérez N. M. Ramakrishnan G. (2014). The reduced genome of the *Francisella tularensis* live vaccine strain (LVS) encodes two iron acquisition systems essential for optimal growth and virulence. PLoS ONE 9:e93558. 10.1371/journal.pone.0093558 24695402 PMC3973589

[B52] Pérez N. Johnson R. Sen B. Ramakrishnan G. (2016). Two parallel pathways for ferric and ferrous iron acquisition support growth and virulence of the intracellular pathogen *Francisella tularensis* Schu S4. Microbiologyopen 5, 453–468. 10.1002/mbo3.342 26918301 PMC4905997

[B53] Ramakrishnan G. Sen B. (2014). The FupA/B protein uniquely facilitates transport of ferrous iron and siderophore-associated ferric iron across the outer membrane of *Francisella tularensis* live vaccine strain. Microbiology 160(Pt 2), 446–457. 10.1099/mic.0.072835-0 24307666 PMC3919536

[B54] Ramakrishnan G. Meeker A. Dragulev B. (2008). Fsle is necessary for siderophore-mediated iron acquisition in *Francisella tularensis* Schu S4. J. Bacteriol. 190, 5353–5361. 10.1128/JB.00181-08 18539739 PMC2493265

[B55] Ramakrishnan G. Sen B. Johnson R. (2012). Paralogous outer membrane proteins mediate uptake of different forms of iron and synergistically govern virulence in *Francisella tularensis tularensis*. J. Biol. Chem. 287, 25191–25202. 10.1074/jbc.M112.371856 22661710 PMC3408188

[B56] Rohmer L. Brittnacher M. Svensson K. Buckley D. Haugen E. Zhou Y.. (2006). Potential source of *Francisella tularensis* live vaccine strain attenuation determined by genome comparison. Infect. Immun. 74, 6895–6906. 10.1128/IAI.01006-06 17000723 PMC1698093

[B57] Rohmer L. Fong C. Abmayr S. Wasnick M. Larson Freeman T. J. Radey M.. (2007). Comparison of *Francisella tularensis* genomes reveals evolutionary events associated with the emergence of human pathogenic strains. Genome Biol. 8:R102. 10.1186/gb-2007-8-6-r102 17550600 PMC2394750

[B58] Salomonsson E. Kuoppa K. Forslund A. L. Zingmark C. Golovliov I. Sjöstedt A.. (2009). Reintroduction of two deleted virulence loci restores full virulence to the live vaccine strain of *Francisella tularensis*. Infect. Immun. 77, 3424–3431. 10.1128/IAI.00196-09 19506014 PMC2715654

[B59] Sen B. Meeker A. Ramakrishnan G. (2010). The *fslE* Homolog, *FTL-0439* (*fupA/B*), Mediates siderophore-dependent iron uptake in *Francisella tularensis* LVS. Infect. Immun. 78:10. 10.1128/IAI.00503-10 20696823 PMC2950358

[B60] Sharma J. Mares C. A. Li Q. Morris E. G. Teale J. M. (2011). Features of sepsis caused by pulmonary infection with *Francisella tularensis* Type A strain. Microb. Pathog. 51, 39–47. 10.1016/j.micpath.2011.03.007 21440052 PMC3090489

[B61] Sjostedt A. (2007). Tularemia: history, epidemiology, pathogen physiology, and clinical manifestations. Ann. N. Y. Acad. Sci. 1105, 1–29. 10.1196/annals.1409.009 17395726

[B62] Stevenson B. Wyckoff E. E. Payne S. M. (2016). *Vibrio cholerae* FeoA, FeoB, and FeoC interact to form a complex. J. Bacteriol. 198, 1160–1170. 10.1128/JB.00930-15 26833408 PMC4800862

[B63] Su J. Yang J. Zhao D. Kawula T. H. Banas J. A. Zhang J. R. (2007). Genome-wide identification of *Francisella tularensis* virulence determinants. Infect. Immun. 75, 3089–3101. 10.1128/IAI.01865-06 17420240 PMC1932872

[B64] Sullivan J. T. Jeffery E. F. Shannon J. D. Ramakrishnan G. (2006). Characterization of the siderophore of *Francisella tularensis* and role of *fslA* in siderophore production. J. Bacteriol. 188, 3785–3795. 10.1128/JB.00027-06 16707671 PMC1482922

[B65] Svensson K. Larsson P. Johansson D. Byström M. Forsman M. Johansson A. (2005). Evolution of Subspecies of *Francisella tularensis*. J. Bacteriol. 187, 3903–3908. 10.1128/JB.187.11.3903-3908.2005 15901721 PMC1112057

[B66] Thieken A. Winkelmann G. (1992). Rhizoferrin: a complexone type siderophore of the *Mucorales* and *Entomophthorales* (Zygomycetes). FEMS Microbiol. Lett. 94, 37–41. 10.1111/j.1574-6968.1992.tb05285.x 1387861

[B67] Thomas-Charles C. A. Zheng H. Palmer L. E. Mena P. Thanassi D. G. Furie M. B. (2013). FeoB-mediated uptake of iron by *Francisella tularensis*. Infect. Immun. 81, 2828–2837. 10.1128/IAI.00170-13 23716605 PMC3719576

[B68] Twine S. Byström M. Chen W. Forsman M. Golovliov I. Johansson A.. (2005). A mutant of *Francisella tularensis* strain SCHU S4 lacking the ability to express a 58-kilodalton protein is attenuated for virulence and is an effective live vaccine. Infect. Immun. 73, 8345–8352. 10.1128/IAI.73.12.8345-8352.2005 16299332 PMC1307091

[B69] Weaver E. A. Wyckoff E. E. Mey A. R. Morrison R. Payne S. M. (2013). FeoA and FeoC are essential components of the *Vibrio cholerae* ferrous iron uptake system, and FeoC interacts with FeoB. J. Bacteriol. 195, 4826–4835. 10.1128/JB.00738-13 23955009 PMC3807486

[B70] Wehrly T. D. Chong A. Virtaneva K. Sturdevant D. E. Child R. Edwards J. A.. (2009). Intracellular biology and virulence determinants of *Francisella tularensis* revealed by transcriptional profiling inside macrophages. Cell. Microbiol. 11, 1128–1150. 10.1111/j.1462-5822.2009.01316.x 19388904 PMC2746821

[B71] Weiss D. S. Brotcke A. Henry T. Margolis J. J. Chan K. Monack D. M. (2007). *In vivo* negative selection screen identifies genes required for *Francisella* virulence. Proc. Natl. Acad. Sci. U.S.A. 104, 6037–6042. 10.1073/pnas.0609675104 17389372 PMC1832217

[B72] Wu X. Ren G. Gunning W. T. III. Weaver D. A. Kalinoski A. L. Khuder S. A.. (2016). FmvB: a *Francisella tularensis* magnesium-responsive outer membrane protein that plays a role in virulence. PLoS ONE 11:e160977. 10.1371/journal.pone.0160977 27513341 PMC4981453

[B73] Zajdowicz S. Haller J. C. Krafft A. E. Hunsucker S. W. Mant C. T. Duncan M. W.. (2012). Purification and structural characterization of siderophore (Corynebactin) from *Corynebacterium diphtheriae*. PLoS ONE 7:e34591. 10.1371/journal.pone.0034591 22514641 PMC3326035

